# Catecholamines in sepsis: pharmacological insights and clinical applications—a narrative review

**DOI:** 10.1186/s44158-025-00241-2

**Published:** 2025-04-03

**Authors:** Jacopo Belfiore, Riccardo Taddei, Giandomenico Biancofiore

**Affiliations:** Anesthesia and Transplant Intensive Care Unit, Department of Anesthesiology and Intensive Care, University of Pisa, Pisa, Italy

**Keywords:** Adrenergic receptors, Toll-like receptors, Cytokines, Endothelial dysfunction, Immunomodulatory effect, Corticosteroids

## Abstract

Catecholamines, essential neurotransmitters and hormones, play a critical role in the body’s physiological response to stress and are pivotal in the management of various clinical conditions, particularly in critical care settings. This narrative review delves into the pharmacological properties of catecholamines, including their mechanisms of action, pharmacokinetics, and pharmacodynamics. Key clinical applications of catecholamines, especially in the cardiovascular and immune systems, are highlighted, emphasizing their roles in modulating heart rate, vascular tone, and immune responses during critical conditions such as sepsis and septic shock. Additionally, the review explores catecholamines’ immunomodulatory effects and their interactions with other therapeutic agents, such as corticosteroids, in the management of septic shock. Further research is suggested to optimize catecholamine usage and improve patient outcomes in critical care settings.

## Introduction

Sepsis and septic shock represent significant global health challenges, with high mortality rates, and they remain leading causes of death in critically ill patients. These conditions arise from a dysregulated host immune response to infection, leading to multi-organ dysfunction and hemodynamic instability [1]. Managing septic shock requires a multifaceted approach, including volume resuscitation, infection control, and the use of vasoactive agents to restore and maintain adequate perfusion pressure [2]. Catecholamines, such as norepinephrine, dopamine, and epinephrine, are pivotal in the management of septic shock and other critical care conditions. Acting as neurotransmitters and hormones, catecholamines play a central role in modulating cardiovascular and immune responses to stress. Their ability to increase cardiac output, restore vascular tone, and regulate inflammatory pathways underscores their importance in addressing the complex pathophysiology of sepsis [2, 3]. Despite their widespread use, catecholamines are not without limitations. Excessive adrenergic stimulation can contribute to adverse effects, including immunosuppression, metabolic disturbances, and organ dysfunction, necessitating a deeper understanding of their mechanisms and clinical applications [4, 5]. Emerging evidence highlights the immunomodulatory potential of catecholamines in sepsis, where the balance between pro- and anti-inflammatory responses is critical. Recent studies have also explored the synergistic use of catecholamines with adjunct therapies, such as corticosteroids, to mitigate endothelial dysfunction and improve outcomes. However, conflicting data in the literature emphasize the need for further investigation into optimizing catecholamine use in sepsis management [4–7]. This narrative review aims to provide a comprehensive overview of the existing evidence on catecholamines in sepsis, focusing on their pharmacological properties, mechanisms of action, and clinical applications. By addressing current knowledge gaps and integrating recent evidence, this work seeks to advance the understanding of catecholamines’ roles in managing septic shock and to propose directions for future research.

### Mechanism of action, pharmacokinetics, and pharmacodynamics of the main catecholamines

The biochemical structure of all catecholamines is characterized by a benzene ring containing two hydroxyl groups, an intermediate ethyl chain, and a terminal amine group. Endogenous catecholamines have two functions: neurotransmitter, when released into the synaptic cleft, and hormonal, when released into the systemic circulation. Catecholamines are derived from the hydroxylation of tyrosine to L- 3,4-dihydroxyphenylalanine (DOPA), which is then converted into dopamine by the enzyme DOPA decarboxylase. Dopamine serves as the precursor to other catecholamines, as it undergoes hydroxylation by dopamine beta-hydroxylase to form norepinephrine, which is then methylated by phenylethanolamine N-methyl transferase to produce epinephrine [8]. Once synthesized, catecholamines are stored in cytosolic granules and released via a calcium-dependent mechanism, which is activated by the action potential of adrenergic synapses in the central nervous system and by sympathetic nervous system action on the adrenal medulla [9]. In the central nervous system, the noradrenergic and dopaminergic systems predominate. Neurons located in the lateral tegmental area and the locus coeruleus are the functional centers of the noradrenergic system, where α1 receptors are most represented. Activation of these receptors modulates a wide range of physiological neuro-sensory responses, including muscle tone maintenance, pain and stress response, circadian rhythm regulation, modulation of autonomic nervous system activity, and cognitive functions. Dopamine is widely synthesized in neurons located in the substantia nigra and the ventral tegmental area and exhibits dose-dependent pleiotropic effects [10, 11].

In the autonomic nervous system, pre-ganglionic axonal fibers originating from the spinal cord converge towards the neurons of the paravertebral chain of the sympathetic nervous system, which, in turn, have post-ganglionic fibers forming a nerve plexus along the main branches of peripheral arterial vessels towards target peripheral organs [12, 13]. The targets of the autonomic nervous system in peripheral organs are the smooth muscle cells in the muscular tunica of “resistance” arteries and epithelial gland cells [14, 15].

Finally, chromaffin cells of the adrenal medulla predominantly release adrenaline and noradrenaline (in a ratio of approximately 80:10 in favor of the former), usually as a physiological response to stress. The corresponding biophysical effect is sympathetic stimulation originating from pre-ganglionic fibers between the seventh and ninth thoracic segments [16].

The plasma half-life of catecholamines is relatively short, typically between 1 and 2 min. This characteristic allows for rapid titration to achieve the desired mean arterial pressure levels [17]. The enzymes responsible for catecholamine metabolism are monoamine oxidase (MAO), an enzyme located in the liver, kidney parenchyma, and synaptic terminals, and catechol-O-methyl transferase (COMT), present in the liver and kidneys. The primary difference between these enzymatic systems is the transformation of the amine group into an aldehyde by MAO and the methylation of the hydroxyl group at the C3 position by COMT [18]. Concurrent administration of MAO inhibitors is a relative contraindication to the use of sympathomimetic drugs [19].

All catecholamines exert their effects on vascular smooth muscle cells and cardiomyocytes by modulating intracellular calcium concentration. Dopamine acts on dopaminergic receptors, while norepinephrine and adrenaline act on adrenergic receptors [20]. These receptors form a complex G-protein-coupled receptor system, consisting of an extracellular N-terminal domain complementary to its agonist molecule, seven transmembrane domains, and an intracellular C-terminal domain associated with a G protein that activates one of three possible enzymatic ligands: adenylate cyclase, phospholipase C, or ion channels for Ca + + and K + conductance [21].

β2 and α2 adrenergic receptors are associated with G proteins that activate the cyclic adenosine monophosphate/protein kinase A (cAMP/PKA) complex [22]. Activation of this enzyme complex leads to the phosphorylation of phospholamban, which in turn induces increased calcium reuptake into the sarcoplasmic reticulum. The reduced availability of cellular calcium results in peripheral vasodilation [23].

Conversely, α1 adrenergic receptors are associated with Gq proteins that activate the inositol 1,4,5-trisphosphate/diacylglycerol (IP3/DAG) pathway. Activation of this signaling pathway is responsible for increasing the intracellular calcium concentration, resulting in peripheral vasoconstriction [24].

Stimulation of β1 adrenergic receptors increases intracellular cAMP, which leads to the activation of calcium channels, resulting in increased intracellular calcium concentration, responsible for the main positive inotropic effects on the heart [25].

Dopaminergic receptors are predominantly distributed in the mesenteric, splanchnic, and renal regions, modulating intracellular calcium concentration via the cAMP pathway as a second messenger. As a precursor to all catecholamines, dopamine can modulate the secretion of adrenaline and noradrenaline, with physiological effects either directly on post-synaptic D1 receptors or indirectly on pre-synaptic D2 receptors. Activation of pre-synaptic D2 receptors regulates catecholamine release. The main difference from adrenergic receptor stimulation lies in the dose-dependent therapeutic response on the smooth muscle cells of peripheral vessels: at intermediate doses, calcium reuptake into the sarcoplasmic reticulum prevails (vasodilation), while at higher doses, intracellular calcium concentration increases (vasoconstriction) [26].

### *Main clinical effects (*Fig. 1*)*

**Fig. 1:**
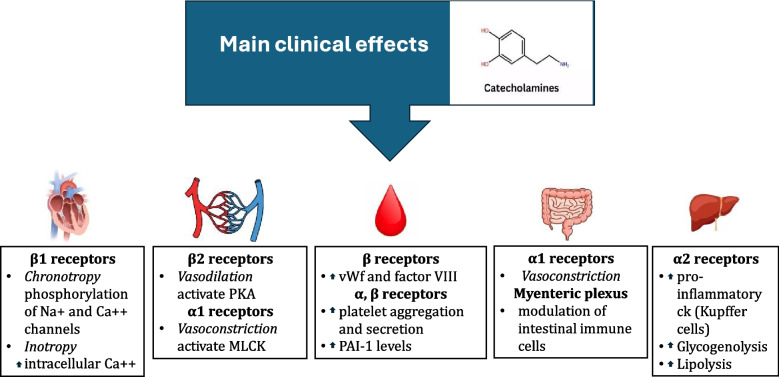
Main clinical effects of catecholamines. PKA: protein kinase A, MLCK: myosin light chain kinase, vWf: von Willebrand factor

#### Cardiovascular system

In general, catecholamines increase cardiac output (CO) through a positive chronotropic effect on heart rate (via β1 receptors) and by increasing preload as a result of venous vasoconstriction, predominantly in the splanchnic region (via α1 receptors). The effects of catecholamines on vascular tone are regulated by two different types of receptors: activation of α1 receptors induces peripheral vasoconstriction, whereas activation of β2 receptors results in vasodilation [27].

#### Chronotropy

Catecholamines modulate heart rate through stimulation of β1 receptors located on the plasma membrane of sinoatrial and atrioventricular node cells. The phosphorylation of Na + and Ca + + channels facilitates the influx of these ions into the cytosol, leading to a positive chronotropic response [28].

#### Inotropy

Activation of β1 receptors on cardiomyocytes increases intracellular Ca + + concentration, which acts on troponin-C, inducing a conformational change in the troponin complex, thereby allowing interaction between actin and myosin filaments. The final effect is an increase in myocardial contractility [29]. Following each depolarization of the cardiomyocyte plasma membrane, Ca + + must be “pumped” out of the cell or back into the sarcoplasmic reticulum through an ATP-dependent process, which requires significant myocardial energy expenditure as the process works against a concentration and electrical gradient. Considering that ATP is also used to reset the myosin heads, the resulting myocardial energy consumption is high [30].

In the peripheral circulation, similarly to cardiomyocytes, contraction of smooth muscle cells in blood vessels is mediated by myosin, which is regulated by phosphorylation promoted by myosin light chain kinase (MLCK). Therefore, peripheral vasodilation and vasoconstriction depend on the activation of MLCK associated with β2 or α1 receptors, respectively [31]. Activation of α1 receptors leads to an increase in calcium concentration and MLCK activation (vasoconstriction), while β2 receptor activation induces an increase in cAMP synthesis, activation of PKA (an enzyme that transmits signals by adding or removing phosphate groups from proteins), and MLCK phosphorylation (vasodilation) [32]. Conversely, certain vascular regions are refractory to catecholaminergic stimuli because they contain relatively low amounts of specific catecholamine receptors. In these areas, the action of locally produced mediators such as adenosine, CO2, and acetylcholine predominates [33]. Finally, while the perfusion of the hepato-splanchnic and musculo-cutaneous regions depends on the balance between mean arterial pressure and peripheral vascular resistance, certain organs have the ability to autoregulate their blood flow within specific ranges of systolic blood pressure (cerebral and renal circulation) [34, 35] or in response to energy consumption (cardiac and cerebral circulation). [34, 36].

#### Gastrointestinal system

Catecholamines modulate splanchnic perfusion by interacting with α adrenergic receptors, allowing the redistribution of blood flow from the intestines to organs with higher energy demands [27, 37]. Moreover, enterocytes can synthesize catecholamines, which can then concentrate in the portal circulation. As a result, Kupffer cells and hepatocytes may be exposed to high concentrations of catecholamines. An increase in norepinephrine concentration in the portal circulation can induce hepatocellular dysfunction through the activation of α2 adrenergic receptors, leading to increased synthesis of pro-inflammatory cytokines by Kupffer cells. Finally, the effect of norepinephrine as a neurotransmitter on the myenteric plexus can modulate the activity of intestinal immune cells, independently of the activation of the autonomic nervous system [38].

#### Metabolism

Regarding energy metabolism, an increase in circulating catecholamines leads to an increase in systemic catabolism, which plays a key role in the body’s physiological response to stress. This phenomenon results in the stimulation of glycogenolysis, leading to increased blood glucose levels, and lipolysis, resulting in increased circulating free fatty acids and ketone bodies, which serve as energy substrates for the brain, heart, and muscle tissues. Lactate derived from anaerobic metabolism in muscle tissue is also utilized as an energy substrate by the brain, liver, heart, and kidneys in response to catecholaminergic stimulation [39].

#### Hemostasis and coagulation

Activation of the sympathetic nervous system can influence the hemostatic system through the release of von Willebrand factor and factor VIII from endothelial cells (β adrenergic stimulation) [40]. Moreover, α and β adrenergic stimulation promotes platelet aggregation and secretion of platelet cytosolic granules [41]. Platelets contain three types of granules that activate the hemostatic process at different levels: lysosomes, which contain various hydrolase enzymes; dense granules, which contain serotonin, histamine, ADP/ATP; and alpha granules, which contain adhesive proteins (vWF, fibronectin, laminin, vitronectin, thrombospondin, fibrinogen), growth factors (PDGF, TGF β, platelet factor 4, thrombospondin), and some coagulation factors (FV, HMWK, C1-inhibitor, FXI, protein S, PAI- 1) [42]. Moreover, catecholamines increase PAI- 1 levels leading to fibrinolysis shutdown [43].

#### Fluid responsiveness

Many authors have demonstrated that the administration of low doses of norepinephrine and phenylephrine infused during the perioperative period in patients undergoing major non-cardiac surgery increases fluid responsiveness, allows for better assessment of blood volume, prevents fluid overload, and facilitates hemodynamic optimization (in terms of stroke volume and cardiac output) by reducing the total amount of fluids administered [44]. α-adrenergic stimulation increases transmural venous pressure, converting unstressed volume (i.e., hemodynamically “unused” volume) into stressed volume, which is “available” to increase preload and, consequently, cardiac output. However, at the same time, the activation of α-adrenergic receptors distributed along the arterial wall of resistance vessels induces vasoconstriction of the arterial vascular bed, reduces tissue perfusion, decreases venous return, and ultimately reduces cardiac output [45]. The final outcome of these two opposing actions depends on the dose of the adrenergic agonist used. Generally, at medium to low doses, the higher density of α-adrenergic receptors on the venous side compared to the arterial side results in a predominance of increased preload due to venoconstriction. The progressive increase in catecholamine dosage leads to an extension of the vasoconstriction effect on both the arterial and venous sides of the circulatory system. In this case, the reduction in peripheral tissue perfusion leaves little room for the recruitment of unstressed volume into the intravascular compartment, resulting in a progressive decrease in preload and, therefore, in cardiac output [46]. The β2-adrenergic stimulation induced by low doses of norepinephrine at the level of hepatic venous vessels reduces their tone, allowing the “spilling over” of splanchnic blood volume into the systemic circulation. The final effect, again, is an increase in preload and cardiac output [47].

### Physiology of catecholamine secretion

The secretion of catecholamines is regulated by the autonomic nervous system and adrenal medulla. In response to stressors such as hypotension, hypoglycemia, or physical and psychological stress, preganglionic sympathetic fibers release acetylcholine, which stimulates chromaffin cells in the adrenal medulla. This stimulation increases intracellular calcium, prompting the fusion of catecholamine-containing granules with the plasma membrane and the release of adrenaline (approximately 80%) and noradrenaline (approximately 20%) into circulation. Secretion is modulated by adrenergic receptor activation through the hypothalamic–pituitary–adrenal axis and sympathetic nervous system, facilitating rapid physiological responses. These include increased heart rate, blood pressure, and blood glucose, critical for managing acute stress and emergency situations [8].

### Alterations in catecholamine secretion during sepsis

Sepsis profoundly disrupts catecholamine secretion through dysregulation of the hypothalamic–pituitary–adrenal axis and the autonomic nervous system. The systemic inflammatory response to sepsis activates the sympathetic nervous system, leading to increased release of endogenous catecholamines such as adrenaline and noradrenaline. Initially, this hyperactivation acts as a compensatory mechanism to sustain cardiac output and peripheral vascular resistance, counteracting septic shock-induced hypotension. However, this response can become maladaptive, contributing to cardiovascular instability and metabolic dysfunction [48]. Pro-inflammatory cytokines, including tumor necrosis factor-alpha (TNF-α) and interleukin- 6 (IL- 6), directly stimulate the adrenal medulla, increasing catecholamine synthesis and secretion [49]. Additionally, activation of toll-like receptors (TLRs) on adrenal chromaffin cells exacerbates catecholamine release [50]. While these mechanisms are initially beneficial in addressing the hemodynamic challenges of sepsis, the sustained activation of the adrenal medulla and sympathetic nerves during sepsis progression can result in “catecholamine refractory shock.” This state is marked by diminished secretion of catecholamines and receptor desensitization, leading to reduced efficacy of endogenous and exogenous catecholamines [51].

### Catecholamines and sepsis: immunomodulatory effects

Sepsis is defined as a potentially fatal syndrome of multiorgan dysfunction caused by a dysregulated host immune response to an infection and is responsible for approximately 20% of global mortality [6]. In recent years, both clinical and basic research have focused heavily on potential therapeutic strategies aimed at treating sepsis-induced immunosuppression [52–54]. The immunoparalysis that occurs during septic shock is characterized by multiple alterations in the immune system, including the reduced expression of HLA-DR isotypes on antigen-presenting cells (APCs), dysregulation of cytokine synthesis resulting in low levels of IL- 6 and TNFα, and a concomitant increase in IL- 10 concentration [55]. The outcome is a diminished ability to clear infectious foci and increased susceptibility to opportunistic pathogens, inevitably leading to higher mortality rates [56]. In this context, norepinephrine, the first-choice vasoactive treatment in septic shock and systemic inflammatory syndromes characterized by hemodynamic instability, has garnered particular interest [51].

Stolk et al. were the first to demonstrate the pleiotropic effects of norepinephrine on the immune system in humans: norepinephrine increases the production of anti-inflammatory cytokines while attenuating the release of pro-inflammatory cytokines and the synthesis of reactive oxygen species (ROS) by circulating leukocytes [57]. The mechanism involved in this process is based on the stimulation of β2 adrenergic receptors, which in turn activate specific protein kinase A, inducing a cAMP-dependent intracellular “cascade” mechanism [4]. Additionally, Stolk et al. demonstrated that the therapeutic use of non-catecholaminergic agonists does not induce any immunomodulatory effect in hosts under experimentally induced endotoxemia, unlike patients treated with catecholaminergic agonists. Conversely, the anti-inflammatory immunomodulatory effects mediated by norepinephrine treatment are countered by the clinical use of non-selective β-blockers or selective β2-blockers: during septic shock, norepinephrine reduces the TNFα/IL- 10 ratio, while β-blockers increase it [57]. Moreover, in a recent in vivo/ex vivo investigation, Miller et al. compared the impact of drugs commonly used in sepsis (i.e., ciprofloxacin, propofol, and norepinephrine), used alone and in combination, on the ex vivo functionality of peripheral blood mononuclear cells drawn from healthy, infected, and septic individuals: the coincubation with each of the drugs reduced cytokine production and phagocytosis in PBMCs isolated from septic patients, and healthy volunteers coincubated with septic serum. This finding further suggested an immunomodulation role for norepinephrine, without addressing the underlying mechanisms [58]. It has also been shown that dysfunction of the energy metabolism in immune system cells is at the root of immunoparalysis during septic shock and contributes to worsening patient outcomes [59, 60]: both glycolysis and oxidative phosphorylation in human circulating monocytes are reduced or suppressed during norepinephrine therapy, potentially contributing to cytokine synthesis modulation in host monocytes [61]. Additionally, norepinephrine reduces the synthesis of reactive oxygen species by monocytes and neutrophil granulocytes, which represent an essential mechanism of bactericidal activity [62].

### Catecholamines and sepsis: effects on vascular permeability and endothelial dysfunction

Altered vascular permeability and endothelial dysfunction are significant pathophysiological changes during sepsis and are often associated with increased clinical severity and poor prognosis [63]: mottled skin, altered peripheral tissue oxygenation, and changes in orthogonal polarization spectral imaging of the sublingual microcirculation are just some signs of persistent microcirculatory alterations that, in sepsis, frequently correlate with multi-organ failure (MOF) and higher mortality risk [64]. Vascular permeability is regulated at the endothelial level by interactions between calcium-associated endothelial cadherin molecules and tight junctions [65]. During sepsis, lipopolysaccharides and other microbial-derived molecules stimulate toll-like receptors (TLRs), activating intracellular signaling pathways mediated by NF-kB and protein kinases. Consequently, a pro-inflammatory endothelial phenotype is expressed, characterized by increased production of pro-inflammatory cytokines, chemokines, pro-coagulant and antifibrinolytic factors, and greater expression of adhesion molecules (I-CAM, V-CAM). At the same time, endothelial cell apoptosis is promoted, and the glycocalyx is damaged, causing altered vascular permeability and interstitial edema [63–65].

Recent evidence has shown in vitro that catecholamines, through β1 and β2 adrenergic stimulation (but not α adrenergic stimulation), can significantly reduce the increased endothelial permeability induced by lipopolysaccharides and all TLR endothelial agonists [66]. Under experimental conditions, these effects of catecholamines have been observed across a wide range of blood concentrations (0.1–100 μM) and occur within minutes. Therefore, it has been hypothesized that catecholamines may restore endothelial protein interactions to reduce permeability and promote the transcriptional regulation of genes involved in microcirculatory permeability function [67]. β Adrenergic stimulation stabilizes actin filaments in the cytoskeleton (via the Gs-cAMP-PKA protein pathway) and activates membrane-associated RhoA kinase, contributing to the maintenance of vascular permeability [67, 68]. If these experimental findings are reproduced and confirmed in clinical trials, they would support the early use of norepinephrine in septic shock patients to limit the incidence and severity of interstitial edema associated with fluid overload, often resulting from aggressive early fluid therapy. Conversely, catecholamines do not appear to reduce cytokine production induced by TLR endothelial agonists, suggesting that their molecular mechanisms differ from those underlying the alteration of vascular permeability induced by the same agonists [67, 68].

### Catecholamines and sepsis: timing and synergy with corticosteroids

Corticosteroids can be used in the treatment of septic shock due to their well-known anti-inflammatory properties, which work by inhibiting the nuclear transcription factor NF-kB, inhibiting the synthesis of many pro-inflammatory cytokines, particularly IL- 1, IL- 6, IL- 8, TNF-α, and TNF-α receptors type 1 and 2 [69]. Additionally, thanks to their inhibitory action on nitric oxide synthase (NOS), corticosteroids can counteract NO-mediated vasodilation caused by endothelial dysfunction characteristic of septic shock [70]. Finally, exogenous corticosteroids are also used as a therapeutic supplement to counteract the deficit of endogenous corticosteroids due to adrenal insufficiency, which is responsible for further hemodynamic instability and manifests in patients with septic shock [71].

To date, the role of corticosteroid therapy remains a subject of debate, especially regarding its potential negative effects on mortality, length of stay, and vasopressor dosage related to its use, due to the immunosuppression, hyperglycemia, and hyponatremia they can induce in this patient category [72]. The four main clinical trials on corticosteroid treatment during septic shock have shown contrasting results. The APROCCHSS study [73] and Annane et al. [74] reported an overall reduction in mortality, while the CORTICUS [75] and ADRENAL [76] studies did not confirm these benefits. However, most clinical trials have shown that the use of hydrocortisone during septic shock is associated with a more rapid resolution of shock [69], improved hemodynamic stability and decrease in catecholamine requirement [77], justifying its use in septic patients receiving vasopressors. The reasons for the heterogeneity in the studies’ outcomes remain unclear. In a study derived by the VANISH trial [78], two transcriptomic sepsis response signatures (SRSs) associated with immune function and outcome in sepsis were identified: septic patients with the immunocompetent SRS2 endotype treated with corticosteroids were found to have poorer survival than those given placebo. SRS endotype at the onset of septic shock appeared therefore to influence response to corticosteroids [79]. On the other hand, contrasting findings were reported by Wong et al. in both pediatric and adult populations, where hydrocortisone exposure tended to be associated with increased mortality in the subgroup of patients identified as “endotype A”, defined by general repression of the 100 endotype-defining genes and showing a relative suppression of adaptive immunity [80, 81]. To further address this issue, the RECORD trial is ongoing, aiming to identify clinical or biological subgroups of septic patients that would share common signatures relevant to corticosteroid responsiveness, and could enable predictive enrichment for future trials and precision therapy for septic shock [82]. With the current evidence, the clinical indication for the introduction of corticosteroids during septic shock is reserved for patients with hemodynamic instability after adequate volume resuscitation has been performed [2, 6]. Despite conflicting opinions in the literature regarding dosage, current guidelines recommend an intravenous dose of hydrocortisone equivalent to 200 mg over 24 h [6]. There are also conflicting evidence regarding the timing of corticosteroid therapy initiation in septic shock [69]; however, the Surviving Sepsis Campaign guidelines suggest initiating corticosteroid use in septic shock patients within four hours of starting vasopressor therapy and with a norepinephrine dosage of at least 0.25 mcg/kg/min [6]. Ammar et al. recently confirmed the benefits associated with early initiation of corticosteroid therapy (within 24 h of shock onset) during septic shock, following adequate volume resuscitation and during vasopressor treatment at a dosage of 0.5–1 mcg/kg/min of norepinephrine equivalent. [69].

## Conclusions

Our review has explored the pharmacological properties and clinical advantages of catecholamines in managing septic shock, particularly their role in modulating endothelial dysfunction, vascular permeability, and immune responses. However, we also emphasized the potential adverse effects associated with catecholamine administration, such as catecholamine-refractory shock and immunoparalysis. Future research should focus on developing therapeutic strategies that optimize catecholamine use while mitigating these adverse effects. Investigating synergistic approaches with other vasoactive and non-vasoactive agents holds promise for reducing the incidence of complications and enhancing the therapeutic efficacy of catecholamines in septic shock. Additionally, the role of personalized medicine, including biomarkers for tailored catecholamine use, and the exploration of adjunct therapies targeting metabolic and immune pathways, represent critical areas for further investigation. These efforts will be pivotal in refining the clinical management of septic shock and improving patient outcomes.

## Data Availability

No datasets were generated or analysed during the current study.
